# Resistance to acetolactate synthase inhibitors is due to a W 574 to L amino acid substitution in the *ALS* gene of redroot pigweed and tall waterhemp

**DOI:** 10.1371/journal.pone.0235394

**Published:** 2020-06-29

**Authors:** Vijay K. Nandula, Darci A. Giacomini, Jeffery D. Ray

**Affiliations:** 1 Crop Production Systems Research Unit, USDA-ARS, Stoneville, Mississippi, United States of America; 2 Department of Crop Sciences, University of Illinois, Urbana, Illinois, United States of America; 3 Crop Genetics Research Unit, USDA-ARS, Stoneville, Mississippi, United States of America; University of Wyoming, UNITED STATES

## Abstract

Several *Amaranthus* spp. around the world have evolved resistance (and cross resistance) to various herbicide mechanisms of action. Populations of redroot pigweed (RRPW-R) and tall waterhemp (TW-R) in Mississippi, USA have been suspected to be resistant to one or more acetolactate synthase (ALS) inhibiting herbicides. Whole plant dose-response experiments with multiple ALS inhibitors, ALS enzyme assays with pyrithiobac, and molecular sequence analysis of ALS gene constructs were conducted to confirm and characterize the resistance profile and nature of the mechanism in the RRPW-R and TW-R populations. Two susceptible populations, RRPW-S and TW-S were included for comparison with RRPW-R and TW-R, correspondingly. The resistance index (R/S; the herbicide dose required to reduce plant growth by 50% of resistant population compared to the respective susceptible population) values of the RRPW-R population were 1476, 3500, and 900 for pyrithiobac, imazaquin, and trifloxysulfuron, respectively. The R/S values of the TW-R population for pyrithiobac, imazaquin, and trifloxysulfuron were 51, 950, and 2600, respectively. I_50_ values of RRPW-S and RRPW-R populations for pyrithiobac were 0.062 and 208.33 μM, indicating that the ALS enzyme of the RRPW-R population is 3360-fold more resistant to pyrithiobac than the RRPW-S population under our experimental conditions. The ALS enzyme of the TW-R population was 1214-fold resistant to pyrithiobac compared to the TW-S population, with the I_50_ values for pyrithiobac of ALS from TW-R and TW-S populations being 87.4 and 0.072 μM, correspondingly. Sequencing of the *ALS* gene identified a point mutation at position 574 of the *ALS* gene leading to substitution of tryptophan (W) residue with a leucine (L) residue in both RRPW-R and TW-R populations. Thus, the RRPW-R and TW-R populations are resistant to several ALS-inhibiting herbicides belonging to different chemical classes due to an altered target site, i.e., ALS. Resistance in *Amaranthus* spp. to commonly used ALS-inhibiting herbicides warrants an integrated weed management scheme incorporating chemical, mechanical, and cultural strategies by growers.

## Introduction

Acetolactate synthase (ALS, EC 4.1.3.18) is the first common enzyme for synthesis of the branched-chain amino acids valine, leucine and isoleucine. ALS inhibiting herbicides belong to five chemical classes: sulfonylureas (SU), imidazolinines (IMI), triazolopyrimidines (TP), sulfonylaminocarbonyltriazolinines (SCT), and pyrimidinylthiobenzoic acids (PTB) [1]. Since their discovery in the early 1980s, ALS inhibitors have been extensively used in many agricultural landscapes (row and horticultural crops, pastures, rangeland, rights-of-way, and forestry) due to their favorable properties such as a highly specific mode of action, absent or negligible mammalian toxicity, low dosage use, as well as broad usability and efficacy.

A major downside to the widespread use of ALS inhibitors has been the rapid and extensive evolution of resistance in several grass and broadleaf weed populations across the world. For example, within 5 years of introduction of chlorsulfuron, the first ALS inhibitor to be commercialized, prickly lettuce (*Lactuca serriola* L.) and kochia [(*Kochia scoparia* (l.) Shrad] populations became resistant [2–4]. As of August 2019, 162 weed species have been documented to be resistant to one or more ALS inhibitors [5]. Among these resistant weed species are several *Amaranthus* spp. including redroot pigweed (*A*. *retroflexus* L.) and tall waterhemp [*A*. *tuberculatus* (Moq.) Sauer].

In the majority of cases of resistance to ALS-inhibiting herbicides the mechanism is by an altered ALS enzyme [4]. A few weed species, including some *Amaranthus* spp., have exhibited nontarget site-based resistance (NTSR) to ALS inhibiting herbicides. For example, ALS inhibitor-resistant tall waterhemp and Palmer amaranth [*A*. *palmeri* (S.) Wats.] populations from Illinois and Kansas, respectively, possessed NTSR or metabolic resistance conferring characteristics [6,7].

Adverse effects of competition and interference from various weed species on the growth and yield of several crops have been well documented in the literature. Competition from redroot pigweed at a density of one plant per meter of crop row, beginning from crop planting, reduced marketable potato [*Solanum tuberosum* L.] tuber yield by 19 to 33% [8]. Corn [*Zea mays* L.] yield was reduced 5% due to interference from redroot pigweed at 0.5 and 4 plants per m of crop row when corn was at 4-leaf stage or earlier and at 4- to 7-leaf stage, respectively [9]. Further, redroot pigweed emergence before sorghum [*Sorghum bicolor* L. Moench] reached a 5.5-leaf growth stage reduced crop yield significantly [10]. In a three-year study, season-long competition from common waterhemp (now synonymous with tall waterhemp [5]) reduced corn yield by 74 and 11% in the second and third years, respectively [11]. Seed yield of soybean [*Glycine max* L. Merr] was reduced from interference of common waterhemp at the VE, V2-V3, and V4-V5 emergence timings of the crop [12].

Previously, *Amaranthus* species such as Palmer amaranth and spiny amaranth (*A*. *spinosus* L.) biotypes from Mississippi, USA have been reported to be resistant to ALS inhibitors [13,14]. In a Mississippi statewide survey for herbicide resistance to ALS inhibitors, a population each of redroot pigweed and tall waterhemp survived pyrithiobac (a PTB herbicide) at a 1X labeled rate. The objectives of this research were to a) characterize the magnitude of resistance to pyrithiobac; b) determine cross resistance to selected ALS inhibitors; and c) elucidate the physiological and molecular mechanism(s) of resistance in the redroot pigweed and tall waterhemp populations. Whole plant dose response experiments with multiple ALS inhibitors, ALS enzyme assays with pyrithiobac, and molecular sequence analysis of ALS gene constructs were conducted.

## Materials and methods

### Seed collection, storage, germination, planting, growth, and herbicide treatment conditions

In late summer of 2009 and 2010 (July and August), seed from more than 200 populations comprising various pigweed species (Palmer amaranth, redroot pigweed, spiny amaranth, and tall waterhemp) was collected across the state of Mississippi from agronomic fields and non-crop areas, air dried, cleaned, and stored at 0 to 10 °C until further use. For each population, seed from 5 to 10 plants within a 10 m circle was combined, maintaining a distance of at least 1.6 km between populations.

Seeds of wild type/susceptible redroot pigweed (RRPW-S) (Azlin Seed Services, Leland, MS, USA), wild type/susceptible tall waterhemp from a wooded area (TW-S, Stoneville, Washington County, MS, 33.44457 N, -90.90238 W), ALS inhibitor-resistant redroot pigweed (RRPW-R, railroad tracks, Jasper County, MS, 31.88658 N, -88.98182 W), and ALS inhibitor-resistant tall waterhemp (TW-R, soybean [*Glycine max* (L.) Merr.], Monroe County, MS, 33.72475 N, -88.44074 W) populations were planted at 1-cm depth in 50-cm by 20-cm by 6-cm plastic trays with drainage holes containing a commercial potting mix (Metro-Mix^®^ 360, Sun Gro Horticulture, Bellevue, WA, USA). Two weeks after emergence, seedlings were transplanted into 6-cm by 6-cm by 6-cm pots containing the above potting mix. Trays and pots were maintained in a greenhouse set to 25/20 °C day/night, 12-h photoperiod under natural sunlight conditions supplemented with high pressure sodium lights providing 400 μmol m^−2^ s^−1^ of light intensity. Plants were fertilized once with a nutrient solution (Miracle-Gro, The Scotts Company LLC, Marysville, OH, USA) containing 200 mg L^-1^ each of N, P_2_O_5_, and K_2_O 1 wk after transplanting and sub-irrigated as needed, thereafter.

All herbicide treatments were applied with a moving nozzle track sprayer (Devries Manufacturing, Inc., Hollandale, MN, USA) equipped with 8002E nozzles (Spraying Systems Co., Wheaton, IL, USA) delivering 190 L ha^-1^ at 280 kPa to plants that were 10-cm tall and at the four- to six-leaf stage. Above ground shoot tissue was collected 3 weeks after treatment, dried in an oven at 60 °C for 72 to 96 h, and weighed. Dry shoot weights are expressed in terms of percent of nontreated control (no herbicide check). All studies were conducted from 2017 to 2018 at the Jamie Whitten Delta States Research Center of USDA-ARS in Stoneville, MS, except partial DNA sequencing performed at University of Illinois, Urbana, IL.

### Pyrithiobac dose response and cross resistance

Plants of RRPW-R and TW-R were treated with pyrithiobac (0, 0.055, 0.11, 0.21, 0.43, 0.85. 1.7, 3.4, and 6.8 kg ai ha^−1^) (Staple^®^LX, FMC Corp., Wilmington, DE, USA), imazaquin (0, 0.14, 0.28, 0.56, 1.12, 2.24, 4.5, 8.9, and 17.9 kg ai ha^−1^) (Scepter^®^, AMVAC Chemical Corp., Los Angeles, CA, USA), and trifloxysulfuron (0, 0.015, 0.031, 0.062, 0.12, 0.25, 0.5, 1.0, and 2.0 kg ai ha^−1^) (Envoke^®^, Syngenta Crop Protection, Greensboro, NC, USA). RRPW-S and TW-S plants were also treated with the same herbicides, but at the following rates: pyrithiobac (0, 0.002, 0.007, 0.03, 0.11, and 0.43 kg ha^−1^), imazaquin (0, 0.002, 0.009, 0.04, 0.14, and 0.56 kg ha^−1^), and trifloxysulfuron (0, 0.0001, 0.0005, 0.002, 0.008, and 0.031 kg ha ^−1^). The respective rates of the various herbicides used represent 0, 1/2X (pyrithiobac), 1X, 2X, 4X, 8X, and 16X (imazaquin and trifloxysulfuron) field rates for the resistant and 0, 1/64X, 1/16X, 1/4X, 1X, and 4X for the susceptible populations. A nonionic surfactant (Induce, Helena Chemical Co., Collierville, TN, USA) was included with all herbicide treatments at 1% v/v. There were five to eight replications per treatment, each replication representing a single plant, and all experiments were repeated once. Pyrithiobac, imazaquin, and trifloxysulfuron belong to PTB, IMI, and SU herbicide families, respectively [1] and are labeled for postemergence weed control in cotton (*Gossypium hirsutum* L.) (pyrithiobac and trifloxysulfuron), and soybean (imazaquin) [15].

### ALS assay

Plants of RRPW-R, RRPW-S, TW-R, and TW-S populations were grown as previously described. ALS enzyme activity from 4- to 6-leaf plants was assayed *in vitro* using procedures similar to previous descriptions [16,17]. Briefly, enzyme/protein was extracted from 4 g of fresh tissue of newly emerged leaves, bulked from 10 to 15 plants, by grinding under liquid nitrogen. Each replication represented an independent extraction from a shoot sample. Herbicide concentrations used to inhibit ALS enzyme activity were 0, 0.1, 1, 10, 100, and 1,000 mM of technical grade pyrithiobac. This assay measured acetoin that was formed from acid decarboxylation of acetolactate. Background acetoin sources were included as controls. The experimental lay out was a completely randomized, factorial design with three replications per treatment (herbicide concentrations). The experiment was conducted two times.

### ALS sequence analysis

Tissue was collected from confirmed resistant and susceptible RRPW-R, RRPW-S, TW-R, and TW-S plants. Genomic DNA was extracted from each sample following a modified CTAB protocol [18] and quality checked on a Nanodrop 1000. The *ALS* gene was amplified using primers specific to the 5’ and 3’ untranslated regions of both species (ALS-5UTR-F: 5’-CTTCAAGCTTCAACAATG and ALS-3UTR-R: 5’-CCTACAAAAAGCTTCTCCTCTATAAG). PCR reactions included approximately 100 ng DNA, 5 μL Taq polymerase (New England Biolabs, Ipswich, MA, USA), 1.0 mM MgCl_2_, 0.2 mM each deoxyribonucleotide triphosphate (dNTP), and 0.1 μM of the forward and reverse primers. The thermocycler protocol was as follows: denaturation for 5 min at 95 C; 34 cycles of 95 C denaturation for 30 s, 50 C primer annealing for 30 s, and 72 C extension for 2 min; final extension step of 5 min at 72 C. Each PCR product was run out on 1% agarose gel and the 2,065 bp band was excised and purified from the gel using a QIAquick Gel Extraction Kit (QIAGEN Inc., Germantown, MD, USA). The purified product was sequenced using an ABI BigDye Terminator v3.1 Cycle Sequencing Kit (Applied Biosystems, Inc., Beverly, MD, USA) using the forward and reverse primers (ALS-5UTR-F; ALS-3UTR-R) as well as a third primer to capture the middle of the *ALS* gene (ALS-F2: 5’- GTATCTTTCTAGGTTGCCTAAACC). The sequenced products were then purified and electrophoresed on an ABI 3730xl Capillary DNA Analyzer by the W.M. Keck Center at the University of Illinois. After trimming low-quality bases using Sequencher 5.4 software (Gene Codes Corp., Ann Arbor, MI, USA), the sequences were aligned and analyzed using CLC Sequence Viewer (QIAGEN Inc., Redwood City, CA, USA).

### Statistical analysis

All experiments were conducted using a completely randomized design. Data were analyzed by ANOVA via the PROC GLM statement using SAS software (version 9.2, SAS Institute, Inc., Cary, NC, USA). No significant experimental effect was observed in repeated experiments; therefore, data from experiments were pooled. Nonlinear regression analysis was applied to fit a sigmoidal 3 parametric logistic curve of the form:

$$y=a/\left(1+{\left(x/x_{0}\right)}^{b}\right)$$ (1)

where, *a* is the upper response limit, *x*_0_ is the GR_50_ (herbicide dose required to cause a 50% reduction in shoot dry weight of test plants) or I_50_ (herbicide concentration required to cause a 50% reduction in ALS enzyme activity), and *b* is the slope of the curve to relate effect of herbicide dose and concentration, *x*, on growth of *Amaranthus* plants and ALS activity, *y*, respectively. The herbicide dose range has been represented in log form for better visualization of response. Equation parameters were computed using SigmaPlot (version 11.0, Systat Software, Inc., San Jose, CA 95110).

## Results

### Pyrithiobac dose response and cross resistance

**Fig 1:**
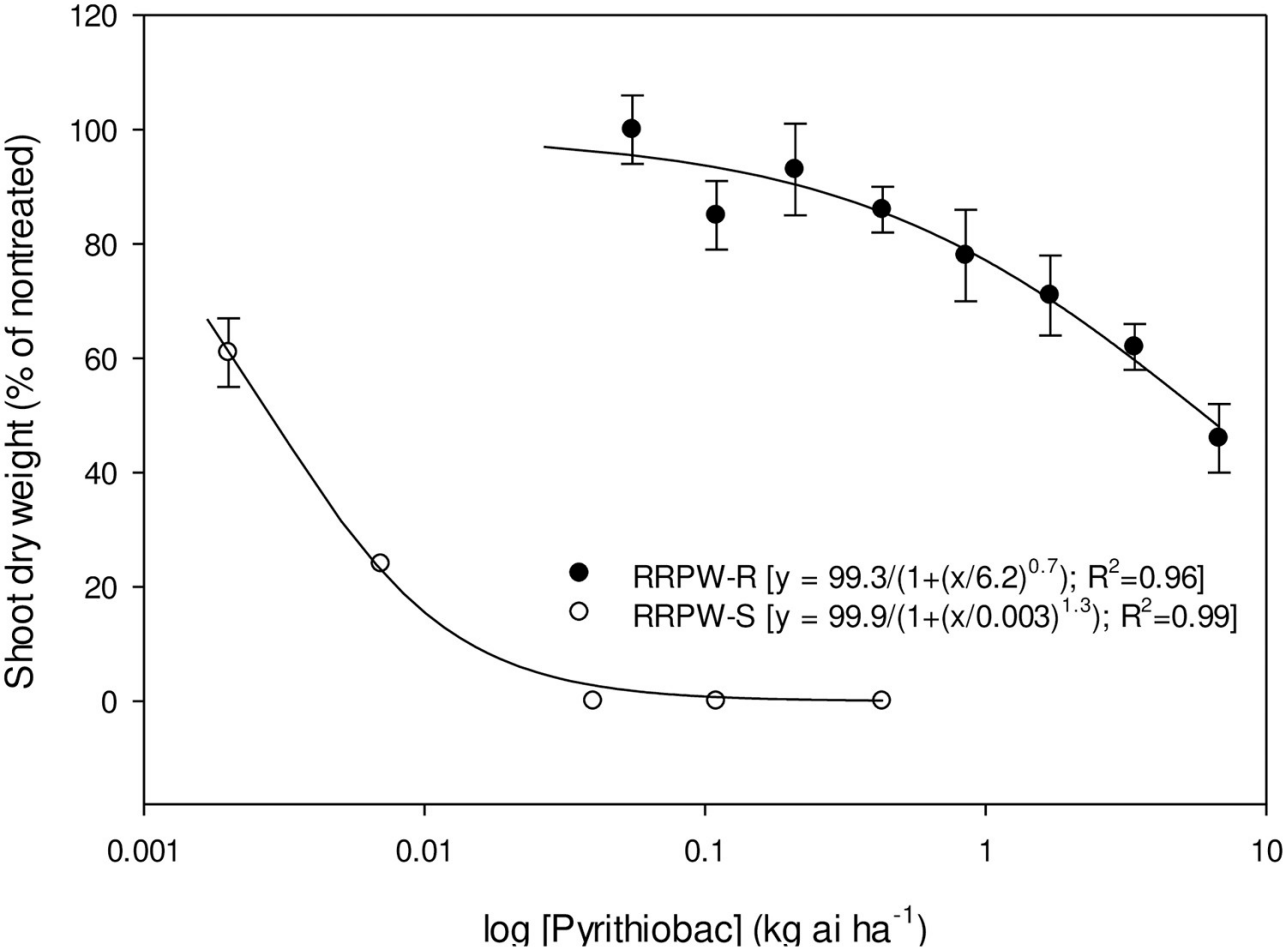
Pyrithiobac dose response on shoot dry weight reduction of ALS-inhibiting herbicide-resistant (RRPW-R) and -susceptible (RRPW-S) *Amaranthus retroflexus* populations from Mississippi 3 wk after treatment. Vertical bars represent standard error of mean.

**Fig 2:**
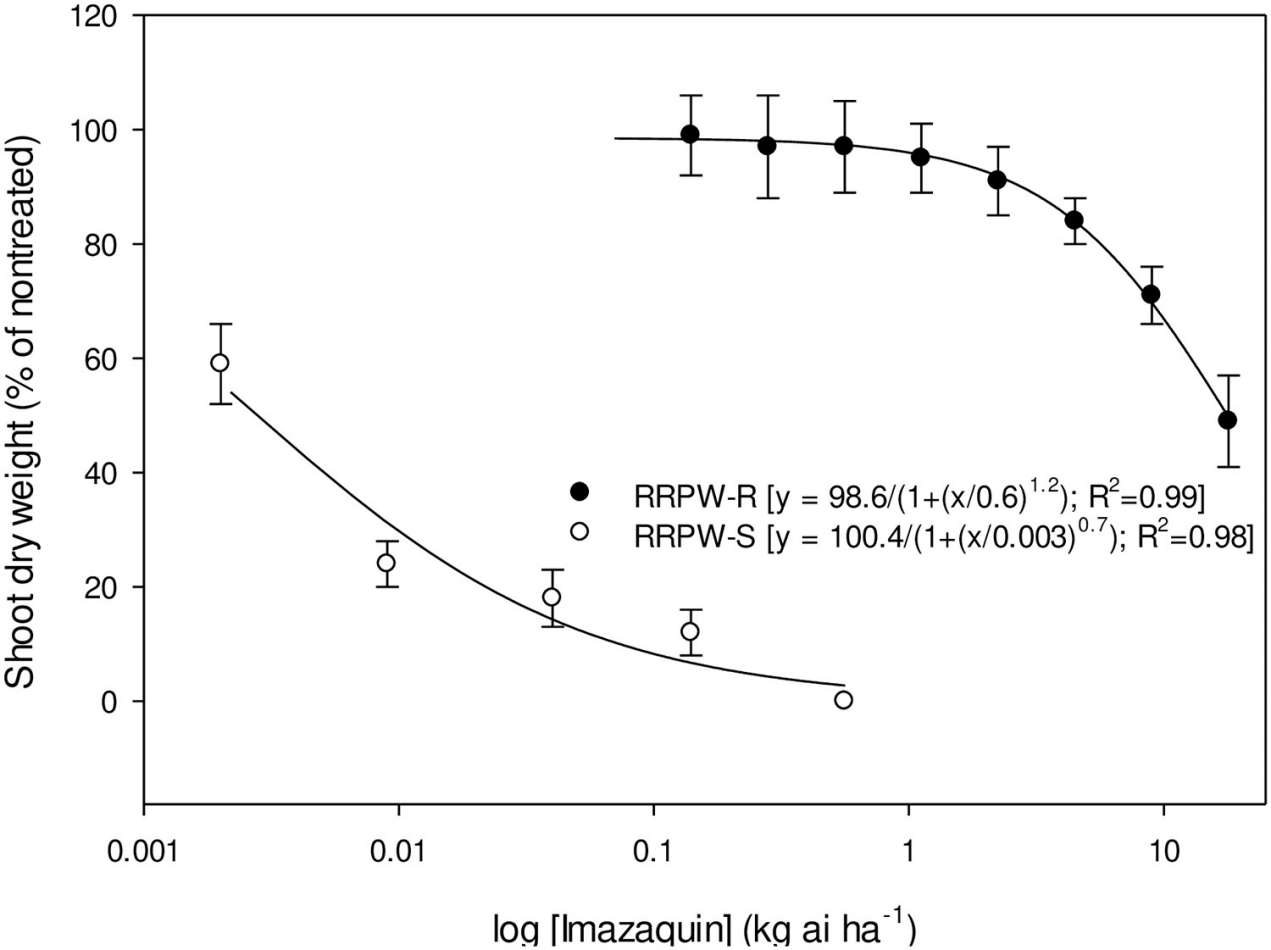
Imazaquin dose response on shoot dry weight reduction of ALS-inhibiting herbicide-resistant (RRPW-R) and -susceptible (RRPW-S) *Amaranthus retroflexus* populations from Mississippi 3 wk after treatment. Vertical bars represent standard error of mean.

**Fig 3:**
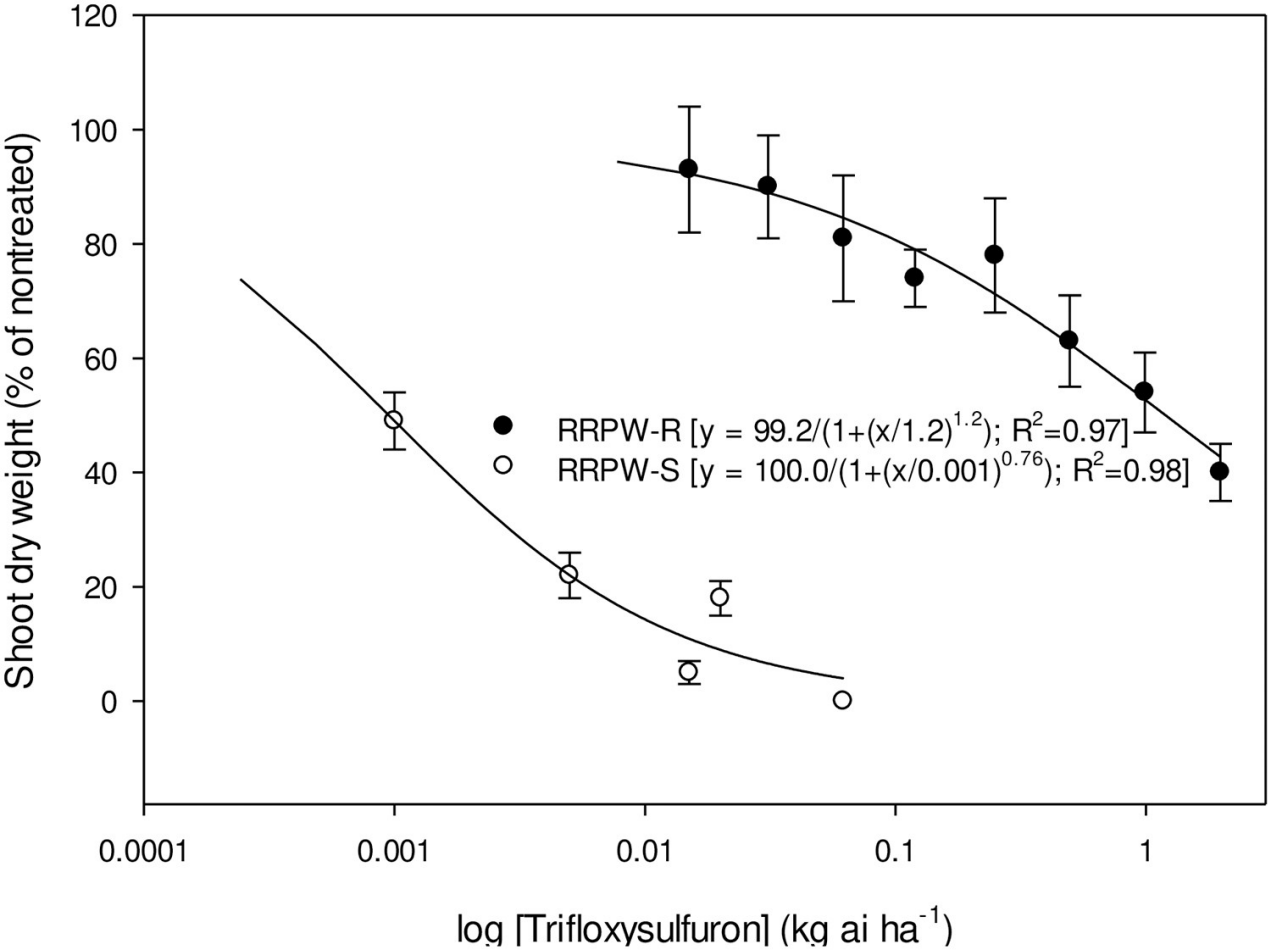
Trifloxysulfuron dose response on shoot dry weight reduction of ALS-inhibiting herbicide-resistant (RRPW-R) and -susceptible (RRPW-S) *Amaranthus retroflexus* populations from Mississippi 3 wk after treatment. Vertical bars represent standard error of mean.

Whole-plant dose response of RRPW-S and RRPW-R populations to pyrithiobac, imazaquin, and trifloxysulfuron is represented in Figs 1–3, respectively. The GR_50_ values (± confidence intervals, CI) of the RRPW-S and RRPW-R populations for pyrithiobac, imazaquin, and trifloxysulfuron were 0.004±0.001, 0.005±0.001, and 0.0001±0.0 kg ha^-1^, and 6.2±1.4, 17.5±3.6, and 0.09±0.005 kg ha^-1^, respectively. Thus, the R/S values of the RRPW-R population were 1476, 3500, and 900 for pyrithiobac, imazaquin, and trifloxysulfuron, respectively.

**Fig 4:**
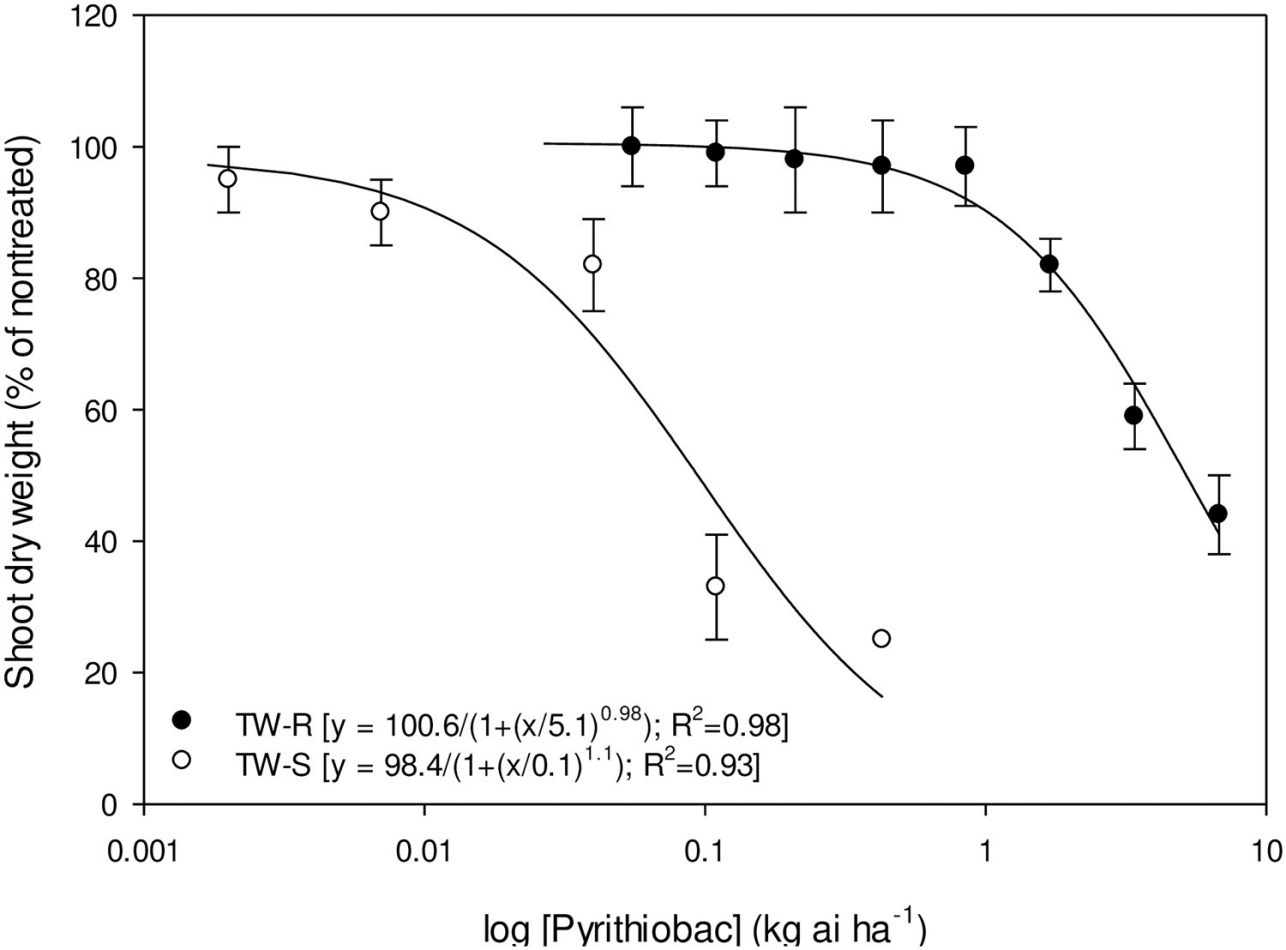
Pyrithiobac dose response on shoot dry weight reduction of ALS-inhibiting herbicide-resistant (TW-R) and -susceptible (TW-S) *Amaranthus tuberculatus* populations from Mississippi 3 wk after treatment. Vertical bars represent standard error of mean.

**Fig 5:**
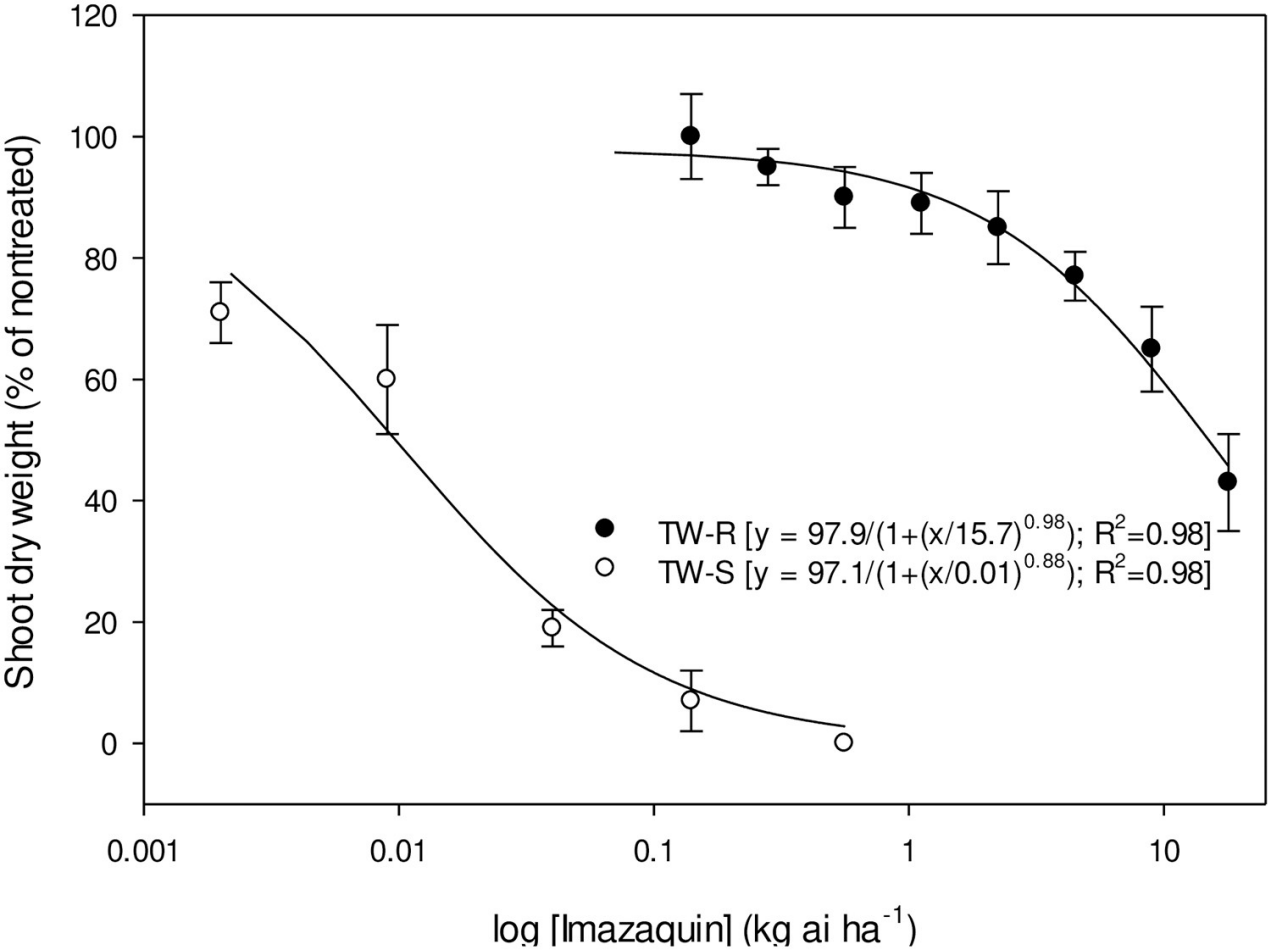
Imazaquin dose response on shoot dry weight reduction of ALS-inhibiting herbicide-resistant (TW-R) and -susceptible (TW-S) *Amaranthus tuberculatus* populations from Mississippi 3 wk after treatment. Vertical bars represent standard error of mean.

**Fig 6:**
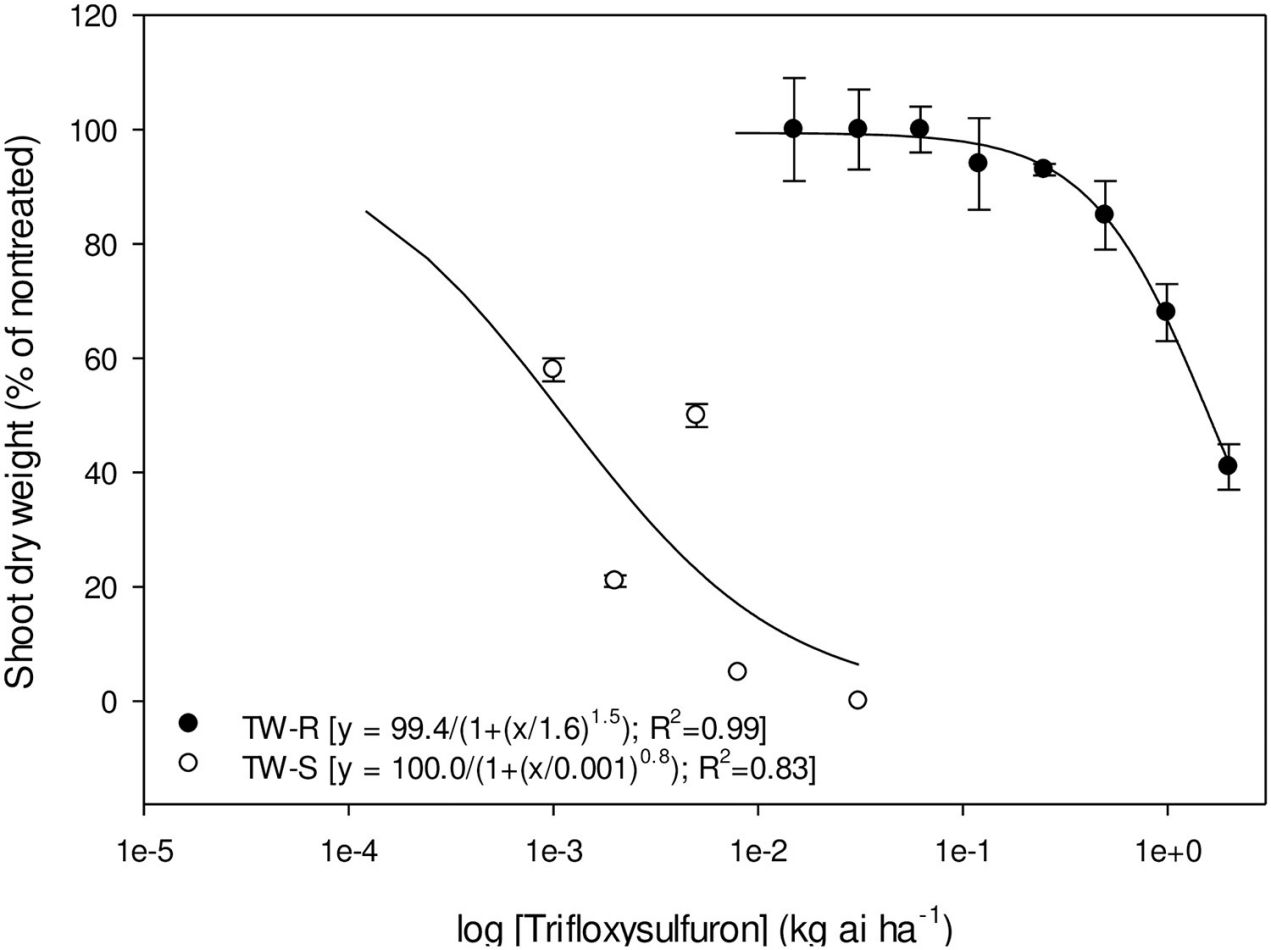
Trifloxysulfuron dose response on shoot dry weight reduction of ALS-inhibiting herbicide-resistant (TW-R) and -susceptible (TW-S) *Amaranthus tuberculatus* populations from Mississippi 3 wk after treatment. Vertical bars represent standard error of mean.

Whole-plant dose response of TW-S and TW-R populations to pyrithiobac, imazaquin, and trifloxysulfuron is represented in Figs 4–6, respectively. The GR_50_ values (±CI) of the TW-S and TW-R populations were 0.09±0.02, 0.012±0.007, and 0.0005±0.0 kg ha^-1^, and 4.6±0.82, 11.4±2.7, and 1.3±0.26 kg ha^-1^ of pyrithiobac, imazaquin, and trifloxysulfuron, respectively. Thus, the R/S values of the TW-R population for pyrithiobac, imazaquin, and trifloxysulfuron were 51, 950, and 2600, respectively.

### ALS assay

**Fig 7:**
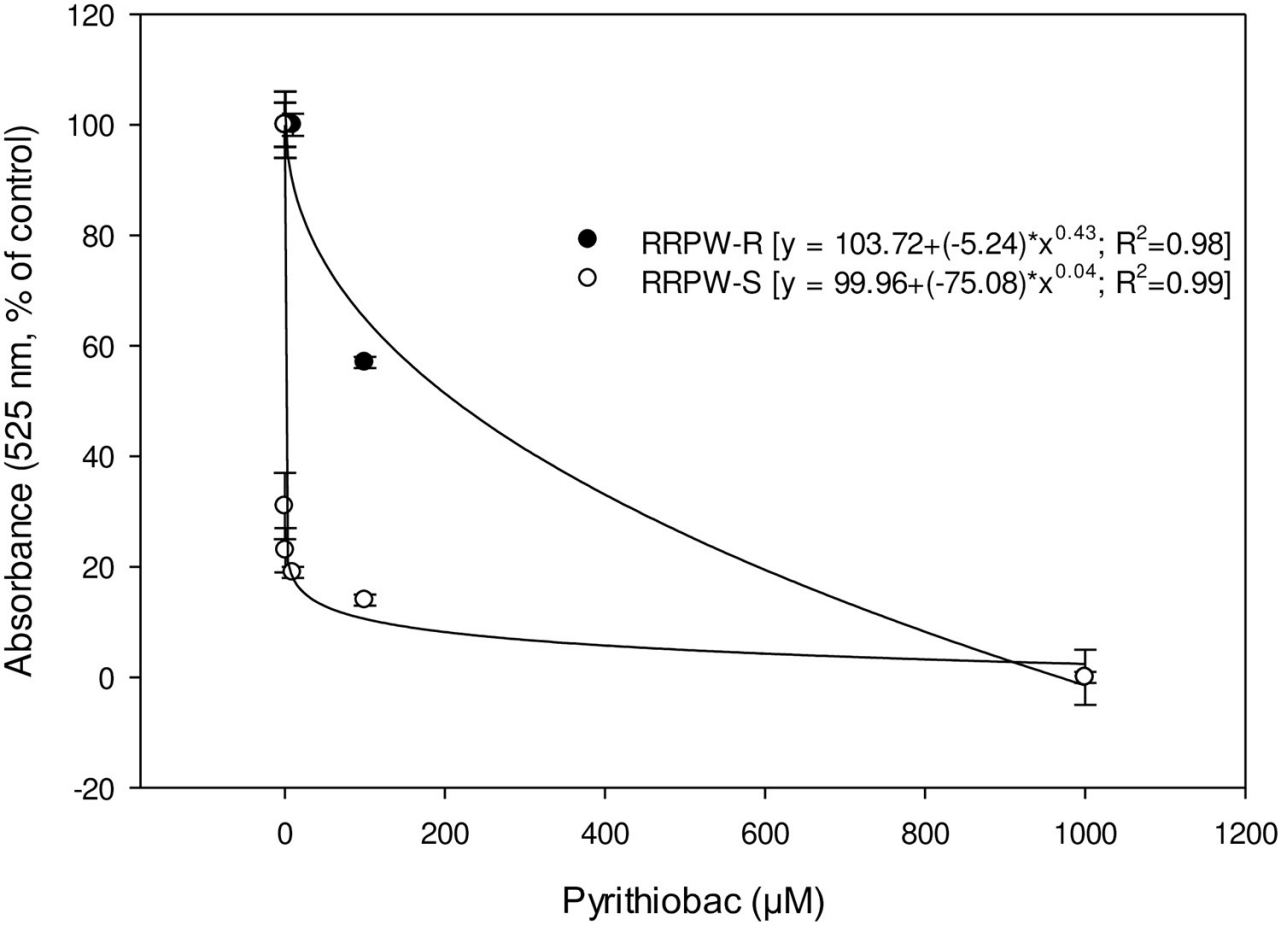
Pyrithiobac dose response ALS enzyme activity of ALS-inhibiting herbicide-resistant (RRPW-R) and -susceptible (RRPW-S) *Amaranthus retroflexus* populations from Mississippi. Vertical bars represent standard error of mean.

**Fig 8:**
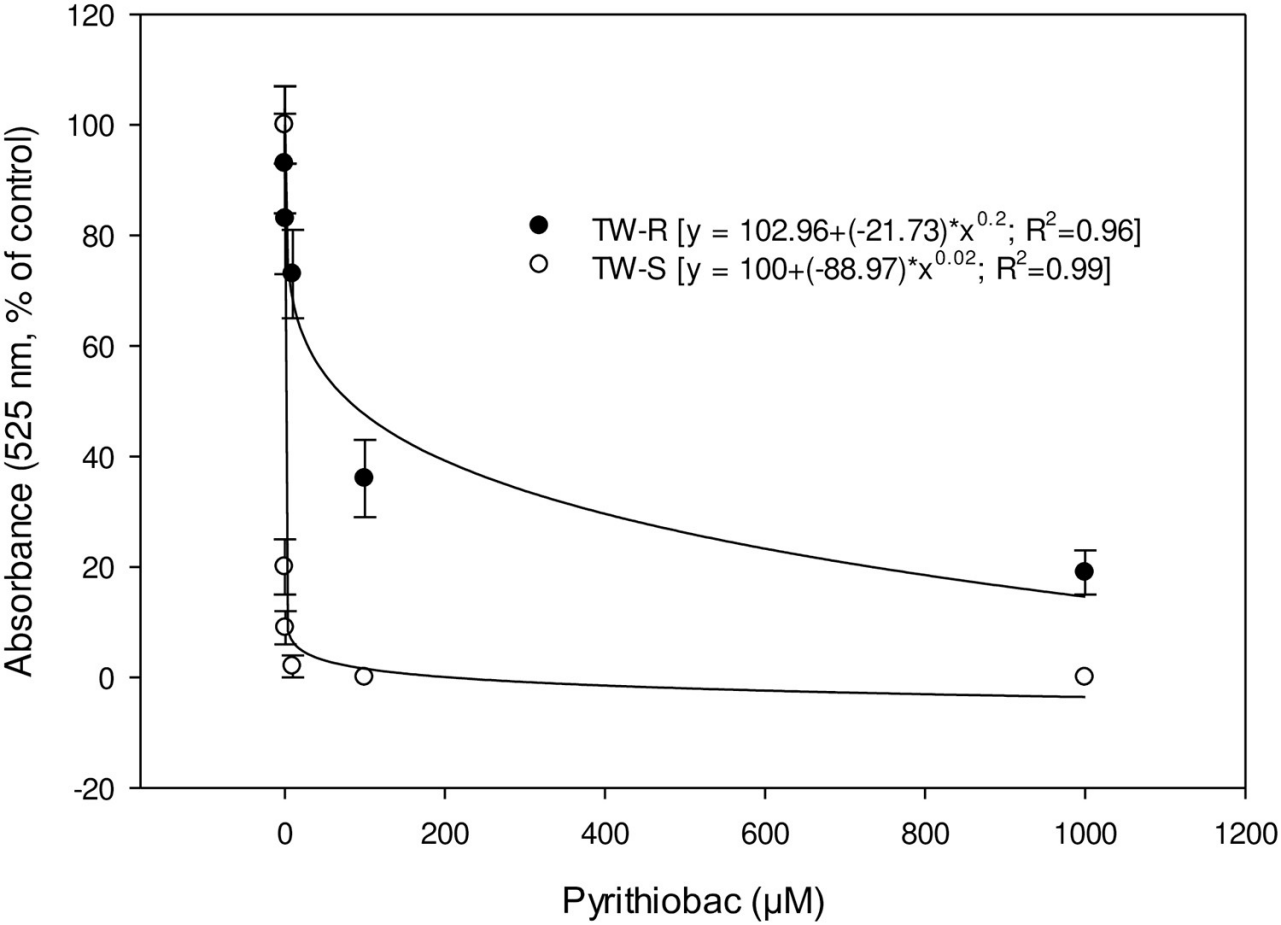
Pyrithiobac dose response on ALS enzyme activity of ALS-inhibiting herbicide-resistant (TW-R) and -susceptible (TW-S) *Amaranthus tuberculatus* populations from Mississippi. Vertical bars represent standard error of mean.

Response of ALS from RRPW-R and RRPW-S to pyrithiobac is represented in Fig 7. I_50_ values (±CI) of RRPW-S and RRPW-R populations for pyrithiobac were 0.062±0.015 and 208.33±12.4 μM, indicating that the ALS enzyme of the RRPW-R population is 3360-fold more resistant to pyrithiobac than the RRPW-S population under our experimental conditions. Response of ALS from TW-R and TW-S to pyrithiobac is represented in Fig 8. The TW-R population was 1214-fold resistant to pyrithiobac compared to the TW-S population, with the I_50_ values (±CI) for pyrithiobac of TW-R and TW-S populations being 87.4±10.5 and 0.072±0.014 μM, correspondingly.

### ALS sequence analysis

Summary of results from sequencing of the *ALS* gene is presented in Table 1. Sequences for RRPW-S (MT495631), RRPW-R (MT495632), TW-S (MT495633), and TW-R (MT495634) have been submitted to GenBank. All plants, five each, of RRPW-R and TW-R populations had the same point mutation at position 574 of the *ALS* gene leading to substitution of tryptophan (W) residue with a leucine (L) residue. All plants were homozygous for the above substitution, except one plant of the RRPW-R population. The RRPW-S and TW-S plants were homozygous for the wildtype allele (W) at the 574 position. All *ALS* residues with known mutations leading to evolved resistance to ALS-inhibiting herbicides, including A122, P197, A205, D376, R377, W574, S653, and G654, were sequenced and analyzed, but only the W574L substitution was detected in the RRPW-R and TW-R populations.

**Table 1:** DNA codons and corresponding amino acids at eight loci on the *ALS* gene, known to have point mutations leading to an altered ALS enzyme, in populations of *Amaranthus retroflexus* (RRPW-R and RRPW-S) and *A*. *tuberculatus* (TW-R and TW-S) that are resistant and susceptible to selected ALS-inhibiting herbicides.

Population	Mutation loci							
	A122	P197	A205	D376	R377	W574	S653	G654
	Codon							
RRPW-S	GCA	CCC	GCT	GAT	CGA	TGG	AGC	GGT
RRPW-R	GCA	CCC	GCT	GAT	CGA	T**T**G	AGC	GGT
TW-S	GCT	CCT	GCT	GAT	CGA	TGG	AGC	GGT
TW-R	GCT	CCT	GCT	GAT	CGA	T**T**G	AGC	GGT
	Amino acid^a^							
RRPW-S	A	P	A	D	R	W	S	G
RRPW-R	A	P	A	D	R	**L**	S	G
TW-S	A	P	A	D	R	W	S	G
TW-R	A	P	A	D	R	**L**	S	G

^a^ Amino acid symbols and corresponding amino acids: A, alanine; D, aspartic acid; G, glycine; L, leucine; P, proline; R, arginine; S, serine; W, tryptophan.

## Discussion

Previously, redroot pigweed accessions/biotypes/populations resistant to one or more ALS-inhibiting herbicides, including those evaluated in this research, have been reported from Brazil, Canada, China, Germany, Israel, Italy, Serbia, and the USA [5,19–21]. The resistance level of the RRPW-R population to pyrithiobac, 1476-fold, is higher compared to resistance factors of 3 to 71 [19] and 7 to 38 [20] reported earlier for redroot pigweed from Brazil. The resistance index of 900 to trifloxysulfuron in the RRPW-R population was higher compared to redroot pigweed populations from Brazil that exhibited R/S values of 23 to 58 in 2014 [19] and 339-fold levels reported in 2019 [20]. The 2019 ALS-inhibiting herbicide resistant redroot pigweed populations from Brazil were also multiple resistant to Photosystem II (PSII) inhibitors [20]. Resistance levels to pyrithiobac in redroot pigweed from other parts of the world have not been clearly documented.

Resistance to imazaquin in redroot pigweed populations has been confirmed in several states in the USA including Arkansas, Maryland, and Pennsylvania (multiple resistance to PSII inhibitors) [5] in addition to our report of 3500-fold resistance. Resistance to imazethapyr, an IMI herbicide like imazaquin, in a redroot pigweed biotype from Italy was 1900-fold, with R/S values ranging between 34 and >500 for several ALS inhibitors [18]. Resistance factors for imazethapyr ranged from 33 to 168 in five redroot pigweed populations from Ontario, Canada [21]. Two of these populations were also cross resistant to thifensulfuron, an SU herbicide, at 270- and 1104-fold higher than a susceptible population.

The ALS enzyme of the RRPW-R population was highly insensitive to pyrithiobac compared to the RRPW-S population, 3360-fold resistant, indicating an altered ALS enzyme as the mechanism of resistance. DNA sequencing analysis provided further evidence corroborating the above mechanism, wherein, a point mutation leading to the replacement of a TGG codon with a TTG codon at the 574 position of *ALS* in the RRPW-R population resulted in the substitution of the amino acid tryptophan with leucine. Similar results were reported in an ALS-inhibiting herbicide resistant redroot pigweed biotype from Italy [5]. In a resistant redroot pigweed population from Ontario, Canada, three mutations A122T, A205V, and W574L, were identified [22]. Resistance-endowing mutations in the *ALS* gene are partially dominant at the minimum, and the resistant gene is spread by seed and pollen due to nuclear-regulated expression [4]. In addition, there has been no fitness cost regarding growth and reproduction in ALS inhibitor-resistant weed species in the absence of selection pressure from herbicides [4].

In addition to the TW-R population characterized here, several occurrences of resistance to ALS-inhibiting herbicides in tall waterhemp have been reported from several states in the USA and the Canadian province of Ontario [5]. Almost all these cases involve cross resistance among several herbicides within or across the five families of ALS-inhibiting mode of action and/or multiple resistance to other herbicidal modes of action [5]. Commonly cited examples include resistance to imazethapyr and thifensulfuron in a biotype from Kansas [23] and a biotype from Illinois with >1000-fold resistance index to imazethapyr and cross resistance to thifensulfuron and flumetsulam [24]. Another report on an Illinois population documented a 130-fold resistance index to imazethapyr [25]. Other reports of tall waterhemp populations that are resistant to ALS-inhibiting herbicides are known but are not summarized herein.

The ALS enzyme from the TW-R population exhibited >1200-fold resistance to pyrithiobac compared to the TW-S population. Similarly, ALS of an Illinois tall waterhemp population was >1900-fold more resistant to imazethapyr than a sensitive population [25]. As discussed earlier, such a response most likely involves an altered ALS enzyme. DNA sequencing analysis indicated presence of a point mutation at the 574 position of TW-R *ALS* leading to a substitution of the tryptophan residue at that location with a leucine. Similar results were reported in an ALS-inhibiting herbicide resistant biotype from Illinois [24]. In other resistant tall waterhemp populations from Illinois, mutations leading to a substitution of serine with threonine or asparagine at position 653 in *ALS* that imparted resistance to imazethapyr and thifensulfuron were confirmed [26].

Tall waterhemp is generally considered a wetland weed [27], whereas Palmer amaranth, a close ‘cousin’, traditionally prefers dry and semi-arid environments [28]. However, present day populations of both species have adapted to diverse environments across the North and South American continents. Both weed species are dioecious in nature, i.e. male and female reproductive organs form on different plants. Endowed with the ability to cross pollinate within [29] as well as across species [14,30–32] transferring herbicide-resistance traits, a fast growth rate, C_4_ plant physiology enabling adaptability to hot and dry conditions, and high fecundity, tall waterhemp and Palmer amaranth have established themselves as two of the most troublesome weeds to manage in row crop production systems. Increasing their management challenge multifold is the ability of tall waterhemp and Palmer amaranth to evolve multiple resistance to more than one unique herbicide modes of action [5]. As an indirect result, other summer annual weed species such as redroot pigweed have become lesser management challenges or have disappeared from row crop growing areas.

## Conclusions

Redroot pigweed and tall waterhemp populations from Mississippi that are highly resistant to pyrithiobac and cross resistant to imazaquin and thifloxysulfuron, all ALS-inhibiting herbicides, have been confirmed. The mechanism of resistance in both weed species has been characterized to be due to an altered ALS enzyme based on ALS enzyme assays and sequencing of the respective *ALS* gene. Public and private land managers must implement a combination of chemical, mechanical, and cultural weed management strategies wherever and whenever feasible to manage herbicide resistant populations such as RRPW-R and TW-R.

## Supporting information

S1 Data (XLSX)

## References

[1] Shaner DL. Herbicide handbook. 10th ed Lawrence, KS: Weed Science Society of America; 2014.

[2] Mallory-Smith CA, Thill DC, Dial MJ. Identification of sulfonylurea herbicide-resistant prickly lettuce (*Lactuca serriola*). Weed Technol. 1990; 4: 163–168.

[3] Primiani M, Cotterman MJC, Saari LL. Resistance of kochia (*Kochia scoparia*) to sulfonylurea and imidazolinone herbicides. Weed Technol. 1990; 4: 169–172.

[4] Tranel PJ, Wright TR. Resistance of weeds to ALS-inhibiting herbicides: what have we learned? Weed Sci. 2002; 50: 700–712.

[5] Heap IM. International Survey of Herbicide Resistant Weeds. http://weedscience.org/. Accessed 29 August 2019.

[6] Guo J, Riggins CW, Hausman NE, Hager AG, Riechers DE, Davis AS, et al Nontarget-site resistance to ALS inhibitors in waterhemp (*Amaranthus tuberculatus*). Weed Sci. 2015; 63: 399–407.

[7] Nakka S, Thompson CR, Peterson DE, Jugulam M. Target site–based and non–target site based resistance to ALS inhibitors in Palmer amaranth (*Amaranthus palmeri*). Weed Sci. 2017; 65: 681–689.

[8] Vangessel MJ, Renner KA. Redroot pigweed (*Amaranthus retroflexus*) and barnyardgrass (*Echinochloa crus-galli*) interference in potatoes (*Solanum tuberosum*). Weed Sci. 1990; 38: 338–343.

[9] Knezevic SZ, Weise SF, Swanton CJ. Interference of redroot pigweed (*Amaranthus retroflexus*) in corn (*Zea mays*). Weed Sci. 1994; 42: 568–573.

[10] Knezevic SZ, Horak MJ, Vanderlip RL. Relative time of redroot pigweed (*Amaranthus retroflexus* L.) emergence is critical in pigweed-sorghum [*Sorghum bicolor* (L.) Moench] competition. Weed Sci. 1997; 45: 502–508.

[11] Steckel LE, Sprague CL. Common waterhemp (*Amaranthus rudis*) interference in corn. Weed Sci. 2004; 52: 359–364.

[12] Steckel LE, Sprague CL. Late-season common waterhemp (*Amaranthus rudis*) interference in narrow- and wide-row soybean. Weed Technol. 2004; 18: 947–952.

[13] Nandula VK, Reddy KN, Koger CH, Poston DH, Rimando AM, Duke SO, et al Multiple resistance to glyphosate and pyrithiobac in Palmer amaranth (*Amaranthus palmeri*) from Mississippi and response to flumiclorac. Weed Sci. 2012; 60: 179–188.

[14] Molin WT, Nandula VK, Wright AA, Bond JA. Transfer and expression of ALS inhibitor resistance from Palmer amaranth (*Amaranthus palmeri*) to an *A*. *spinosus* × *A*. *palmeri* hybrid. Weed Sci. 2016; 64: 240–247.

[15] Mississippi State University. Weed control guidelines for Mississippi. Mississippi State, MS: Mississippi State University; 2019.

[16] Nandula VK, Messersmith CG. Mechanism of wild oat (*Avena fatua* L.) resistance to imazamethabenz-methyl. Pestic Biochem Physiol. 2000; 68: 148–155.

[17] Ray TB. Site of action of chlorsulfuron. Plant Physiol. 1984; 75: 827–831. 10.1104/pp.75.3.827 16663712 PMC1067001

[18] Doyle JJ, Doyle JL. A rapid DNA isolation procedure for small quantities of fresh leaf tissue. Phytochem Bull. 1987; 19: 11–15.

[19] Francischini A, Constantin J, Oliveira RS Jr, Santos G, Franchini LHM, Biffe DF, Resistance of *Amaranthus retroflexus* to acetolactate synthase inhibitor herbicides in Brazil, Planta Daninha. 2014; 32: 437–446.

[20] Francischini A, Constantin J, Oliveira RS Jr, Takano HK, Mendes RR. Multiple-and cross-resistance of *Amaranthus retroflexus* to acetolactate synthase (ALS) and photosystem II (PSII) inhibiting herbicides in preemergence. Planta Daninha. 2019; 10.1590/s0100-83582019370100026.

[21] Ferguson GM, Hamill AS, Tardif FJ. ALS inhibitor resistance in populations of Powell amaranth and redroot pigweed. Weed Sci. 2001; 49: 448–453.

[22] McNaughton KE, Letarte J, Lee EA, Tardif F. Mutations in ALS confer herbicide resistance in redroot pigweed (*Amaranthus retroflexus*) and Powell amaranth (*Amaranthus powellii*). Weed Sci. 2005; 53: 17–22.

[23] Horak MJ, Peterson DE, Biotypes of Palmer amaranth (*Amaranthus palmeri*) and common waterhemp (*Amaranthus rudis*) are resistant to imazethapyr and thifensulfuron. Weed Technol. 1995; 9: 192–195.

[24] Foes MJ, Liu L, Tranel PJ, Wax LM, Stoller EW. A biotype of common waterhemp (*Amaranthus rudis*) resistant to triazine and ALS herbicides. Weed Sci. 1998; 46: 514–520.

[25] Sprague CL, Stoller EW, Wax LM, Horak MJ. Palmer amaranth (*Amaranthus palmeri*) and common waterhemp (*Amaranthus rudis*) resistance to selected ALS-inhibiting herbicides. Weed Sci. 1997; 45: 192–197.

[26] Patzoldt WL, Tranel PJ. Multiple ALS mutations confer herbicide resistance in waterhemp (*Amaranthus tuberculatus*). Weed Sci. 2007; 55: 421–428.

[27] Trucco F, Tatum T, Rayburn AL, Tranel PJ. Out of the swamp: Unidirectional hybridization with weedy species may explain the prevalence of *Amaranthus tuberculatus* as a weed. New Phytol. 2009; 184: 819–827. 10.1111/j.1469-8137.2009.02979.x 19659658

[28] Ward SM, Webster TM, Steckel LE. Palmer amaranth (*Amaranthus palmeri*): A review. Weed Technol. 2013; 27: 12–27.

[29] Liu J, Davis AS, Tranel PJ. Pollen biology and dispersal dynamics in waterhemp (*Amaranthus tuberculatus*). Weed Sci. 2012; 60: 416–422.

[30] Wetzel DK, Horak MJ, Skinner DZ, Kulakow PA. Transferal of herbicide resistance traits from *Amaranthus palmeri* to *Amaranthus rudis*, Weed Sci. 1999; 47: 538–543.

[31] Trucco F, Tatum T, Tranel PJ. *Amaranthus hybridus* can be pollinated frequently by *A*. *tuberculatus* under field conditions, Heredity 2005; 94: 64–70. 10.1038/sj.hdy.6800563 15316559

[32] Nandula VK, Wright AA, Bond JA, Ray JD, Eubank TW, Molin WT. EPSPS amplification in glyphosate-resistant spiny amaranth (*Amaranthus spinosus*): a case of gene transfer via interspecific hybridization from glyphosate-resistant Palmer amaranth (*Amaranthus palmeri*), Pest Manag Sci. 2014; 70: 1902–1909. 10.1002/ps.3754 24497375

